# Gold Nanoparticle-Based Colorimetric Sensors: Properties and Application in Detection of Heavy Metals and Biological Molecules

**DOI:** 10.3390/s23198172

**Published:** 2023-09-29

**Authors:** Sri Agung Fitri Kusuma, Jacko Abiwaqash Harmonis, Rimadani Pratiwi, Aliya Nur Hasanah

**Affiliations:** 1 Department of Pharmaceutical Biology, Faculty of Pharmacy, Universitas Padjadjaran, Jalan Raya Bandung-Sumedang KM 21 Jatinangor, Bandung 45363, Indonesia; 2 Department of Pharmaceutical Analysis and Medicinal Chemistry, Faculty of Pharmacy, Universitas Padjadjaran, Jalan Raya Bandung-Sumedang KM 21 Jatinangor, Bandung 45363, Indonesia; jacko19001@mail.unpad.ac.id (J.A.H.); rimadani.pratiwi@unpad.ac.id (R.P.)

**Keywords:** gold nanoparticles, colorimetric sensor, synthesis, heavy metals, biological molecules

## Abstract

During the last decade, advances have been made in nanotechnology using nanomaterials, leading to improvements in their performance. Gold nanoparticles (AuNPs) have been widely used in the field of sensor analysis and are also combined with certain materials to obtain the desired characteristics. AuNPs are commonly used as colorimetric sensors in detection methods. In developing an ideal sensor, there are certain characteristics that must be met such as selectivity, sensitivity, accuracy, precision, and linearity, among others. Various methods for the synthesis of AuNPs and conjugation with other components have been carried out in order to obtain good characteristics for their application. AuNPs can be applied in the detection of both heavy metals and biological molecules. This review aimed at observing the role of AuNPs in its application. The synthesis of AuNPs for sensors will also be revealed, along with their characteristics suitable for this role. In the application method, the size and shape of the particles must be considered. AuNPs used in heavy metal detection have a particle size of around 15–50 nm; in the detection of biological molecules, the particle size of AuNPs used is 6–35 nm whereas in pharmaceutical compounds for cancer treatment and the detection of other drugs, the particle size used is 12–30 nm. The particle sizes did not correlate with the type of molecules regardless of whether it was a heavy metal, biological molecule, or pharmaceutical compound but depended on the properties of the molecule itself. In general, the best morphology for application in the detection process is a spherical shape to obtain good sensitivity and selectivity based on previous studies. Functionalization of AuNPs with conjugates/receptors can be carried out to increase the stability, sensitivity, selectivity, solubility, and plays a role in detecting biological compounds through conjugating AuNPs with biological molecules.

## 1. Introduction

During the last decade, advances have been made in nanotechnology using nanomaterials, leading to improvements in their performance. Nanomaterials have characteristic properties that are attributed to their small size and quantum effects. Nanomaterials have special physical and chemical properties such as fluorescence emission, surface-to-volume ratio, photothermal effect, and optical properties that are different from their bulky counterparts, etc. [1,2]. Over the past decade, advances have been made in this area with the incorporation of nanomaterials into chemical and biosensors, greatly improving their performance [3]. Nanoparticles of noble metals such as gold (Au), silver (Ag), and copper (Cu) [4,5,6] have been the subject of intense research in recent decades because of their size- and shape-dependent optical, electrical, chemical, and biological properties [7,8]. Among these metal nanoparticles, gold ones are among the current concerns of researchers. Gold is the earliest metal element studied and has three valence states: Au^0^, Au^+^, and Au^3+^. Gold nanomaterials are one of the most stable materials [9]. Furthermore, compared to oxides or general carbon-based compounds, or even other nanoparticles, several previous studies have shown that AuNPs are relatively harmless [10,11]. AuNPs have been widely used in many fields and can be fabricated using a variety of components. They have been proven to be useful for imaging [12], cancer therapy [13], drug delivery [14], and chemical and biological sensing [15]. AuNPs as a colorimetric sensor can be used in heavy metal detection for Hg^2+^ [16], Pb^2+^ [17], Ni^2+^ and Zn^2+^ [18], As^3+^ [19], Cd^2+^ [20], and Cr^3+^ [21] as well as biological molecules such as spermine and spermidine [22], quinalphos [23], amyloid-β oligomers [24], and histidine [25].

In developing an ideal sensor, there are certain characteristics that must be met such as selectivity, sensitivity, accuracy, precision, and linearity, among others [26]. AuNPs can be used to establish optical sensing technologies that utilize aggregation (or deaggregation) of the nanoparticles induced by the formation of non-covalent or covalent bonds with target substances. The aggregation of AuNPs turns a solution of AuNPs from wine red to blue [27,28]. Sensors based on a colorimetric approach are significant; their ideal characteristics need to be analyzed. However, there are drawbacks to using AuNPs, for example, the size and shape of the AuNPs must be adjusted to the purpose of the application so that the optimal results can be achieved because differences in the size and shape will affect their properties and distribution [29].

There have been many reviews of AuNPs that have discussed the method of synthesis; one of the most commonly used is the Turkevich method, which is included in chemical synthesis methods [30]. However, there are other synthesis methods that have been used such as physical and biological synthesis [31]. In detection methods, AuNPs have been used in chemical and biological sensing applications [32]. In addition, one review has explained the use of AuNPs in food and environmental safety evaluations [33]. None of the published reviews have discussed which properties of AuNPs are suitable for the detection of certain analytes such as heavy metals and biological molecules. This review intends to identify the role of AuNPs and their application in both chemical and biological sensors and for the colorimetric detection of those analytes. The synthesis of AuNPs as sensors will also be revealed, along with the chemical and physical properties that make them suitable for the role. The materials or journals used in this review were taken from the last 10 years, which keeps them relevant to the current situation.

## 2. Colorimetric Sensors

**Figure 1:**
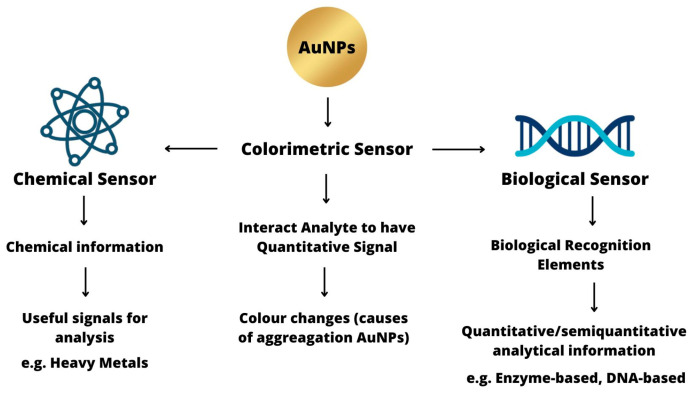
Colorimetric sensors based on AuNPs.

A sensor consists of a chemical or biological receptor that can interact specifically with the target analyte and a transducer that converts the recognition process into a quantitative signal [34]. Chemical and biological sensors are most commonly used to monitor and detect the physicochemical properties of a substance [35]. The colorimetric analysis of AuNPs is related to changes in the color and optical properties caused by the aggregation of AuNPs and changes in particle morphology [36]. Colorimetric sensors are used for instant detection of the analyte and show color changes that can be detected with the naked eye [26]. AuNPs can be used as probes for colorimetric analysis due to their unique size and distance dependent surface plasmon resonance (SPR) properties, which can exhibit observable color changes upon aggregation [27,37]. Colorimetric sensors based on AuNPs use interparticle plasmon coupling to detect the analyte. The aggregation of AuNPs caused by an analyte causes a red shift in the SPR absorption band, which is seen as a red-to-blue color change [36]. In brief, colorimetric sensors based on AuNPs are as shown in Figure 1.

### 2.1. Chemical Sensors

A chemical sensor is a device that converts chemical information or signals, ranging from the concentration of a particular sample component to the analysis of its overall composition, into signals useful for analysis [35]. The chemical signal is produced by a selective interaction between a sensing material placed in the target analyte and sensor. In a chemical sensor, this consists of a sensing element and a transducer [38].

Chemical species such as heavy metals can be harmful to the body and the environment because of their toxic nature, even in trace amounts [39]. AuNPs as colorimetric sensors have appeared as an alternative for the detection of heavy metals [16,17,18,19,20,21]. AuNPs can be conjugated with other compounds to provide better stability, functionality, and biocompatibility [40,41]. In the detection process, AuNPs with conjugated compounds will bind to form aggregation with the detected analyte [42]. The aggregation of AuNPs results in a distinct red-shift in the UV–Vis spectrum and a distinct red-to-blue color change in the colorimetric sensor. In the aggregation mechanism, analytes can be rapidly detected either by visualizing the sensor’s color change or by measuring the UV–Vis spectrum [42].

### 2.2. Biological Sensors

Biological sensors (biosensors) have two aspects, namely the recognition component and transducer, which has a role in offering selectable quantitative or semiquantitative analytical information [43]. Biosensors such as enzyme-based [44], tissue-based [45], immunosensors [46], DNA biosensors [47], thermal [48], and piezoelectric biosensors [49] can sense biochemical compounds. Biosensors have been used in various fields such as microbial detection [50], the food industry [51], medical field [52], and marine sector [53]. Generally, biosensors typically have three parts: (1) the recognition component that reacts to one or more analytes among different compounds [54], (2) the transducer, which converts a generated response based on the resulting reaction between the analytes and the specific biological layer into an optical or electric signal [55], and (3) the electrical system that captures the signal, amplifies it, and records it for data display [56]. For the detection of biological molecules such as proteins or enzymes, there are various conventional methods that can be used such as gas chromatography (GC) [57], high-performance liquid chromatography (HPLC) [58], fluorescence spectrometry [59], and other related technologies. However, those methods have significant limits due to their reliance on chemical and physical principles. Additionally, these methods are expensive, labor-intensive, and have a limited sensitivity range [60].

Noble metal-based nanomaterials have emerged as powerful tools for biosensors with high sensitivity and specificity [42]. Nanomaterials improve the mechanical, optical, and magnetic properties of biosensors [50]. The detection of a biological sensor is based on an aggregation mechanism or anti-aggregation mechanism. These mechanisms have been verified via Fourier transform infrared (FTIR), Ultraviolet–Visible (UV–Vis) spectra, zeta potential, transmission electron microscopy (TEM), and dynamic light scattering (DLS) [42].

## 3. AuNPs

AuNPs are small gold particles with diameters between 1 and 100 nm, also called colloidal gold when dispersed in water [61]. AuNPs are highly stable nanomaterials and have been extensively studied and applied [62]. Currently, the size, shape, and composition of these nanoparticles have influenced their effectiveness in a variety of applications. The most popular method for generating monodisperse particles with purposefully variable sizes and shapes is through the solution phase synthesis of AuNPs [63].

### 3.1. Synthesis

Generally, nanoparticles have different characteristics. AuNPs are metal nanoparticles that can produce different characteristics depending on the method of synthesis. AuNPs can be synthesized using physical, chemical, and biological methods [64,65,66]. These physical, chemical, and biological techniques can be broadly divided into ‘bottom–up’ and ‘top–down’ approaches [67]. Top–down processes use various physical and chemical techniques to reduce bulk materials into nanoscale particles. In contrast, the bottom–up approach moves from small specific particles to nanomaterials [68]. Each synthesis method is compared in Table 1.

**Figure 2:**
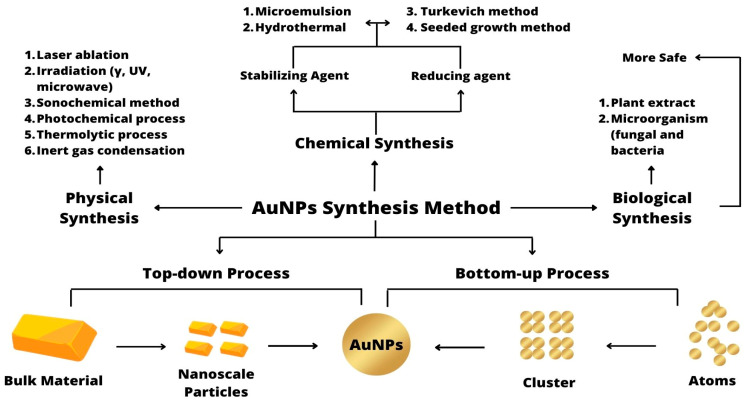
AuNP synthesis methods.

AuNP synthesis can be carried out with one of the three existing synthesis methods as needed. Each method has its own function and advantages. In brief, the AuNP synthesis methods are shown in Figure 2.

#### 3.1.1. Physical Synthesis

Physical methods for the synthesis of AuNPs include laser ablation, γ-irradiation, UV-irradiation, sonochemical methods, microwave irradiation, thermolytic processes, and photochemical processes [72,73,74]. Among the physical methods, laser ablation is the most commonly used method for the synthesis of AuNPs. Laser ablation has been used as a synthesis method mainly due to the unique surface chemistry produced on the AuNPs reduced with laser-assisted wavelengths. The surfaces of the particles produced by the laser are partially oxidized and act as electron acceptors. Therefore, these surfaces can be easily attached to molecular entities containing electron-donating groups such as thiols [75]. An example of this synthesis method has been applied to the analysis of anticancer activity [64].

#### 3.1.2. Chemical Synthesis

In chemical synthesis, there are two phases, namely reduction by a reducing agent and stabilization of the resulting nanoparticles using a suitable stabilizer [76]. The reducing agent provides electrons to reduce the gold ions Au^+^ and Au^3+^ to Au^0^, which is the electrical state of the nanoparticles. In contrast, the agents stabilize the nanoparticles against aggregation by providing a repulsive force that then controls the growth rate, final size, or geometric shape of the nanoparticles. It is possible that the stabilizer is the same molecule that acts as the reducing agent [62].

There are several methods of chemical synthesis such as microemulsion, hydrothermal synthesis, the Turkevich method, and the seeded growth method [77,78]. The most common technique that has been used to synthesize AuNPs is the chemical reduction of gold chloride with sodium citrate by Turkevich et al. (1951) [79]. Sodium citrate acts as both a reducing and capping agent in this synthesis process. During the reduction of trivalent gold (Au^3+^) to zero valent gold (Au^0^), citrate ions are adsorbed on the surface of AuNPs, subsequently imparting a negative charge and stability. There are factors that will affect the size of the AuNPs produced such as the ratio and pH of the citrate and temperature in the synthesis process [65,80]. The citrate method can be used to obtain a wide range of AuNP sizes that can be used for a variety of potential applications [65]. The other critical factors that need to be considered are the storage conditions of the AuNPs and the absence of citrate and non-AuNP components in solution. Regarding storage, AuNPs will be more stable without aggregation in cold storage (4 °C) than at room temperature because they show aggregation over time [81]. The non-AuNPs and excess citrate components must be characterized and removed prior to use [82].

#### 3.1.3. Biological Synthesis

Physical and chemical approaches are the major methods to synthesize AuNPs. A major drawback of these synthesis methods is the use of hazardous materials [31]. The biological synthesis of AuNPs has emerged as a perfect alternative and environmentally friendly approach, making the process viable, safer, and cheaper [83]. Synthesis using cells from microorganisms and plants is the most common method of AuNP biosynthesis [84]. An example of this synthesis method has been applied to the analysis of antifungal action [66].

### 3.2. Properties/Characteristics

For further usage, the produced AuNPs must be suitably characterized. The characterization of nanoparticles is a complex and critical parameter that cannot be measured by metrics such as the chemical composition and concentration. This is due to nanoparticles having unique physical and chemical properties that demand exceptional parameters to characterize them, unlike conventional chemicals [62,76]. The specific optical and physical properties of AuNPs are mainly caused by differences in their size, shape, colloidal stability, and morphology. Therefore, comprehensive characterization using various techniques for the quality control of the produced AuNPs is of great importance [85].

AuNPs are available commercially in various sizes (1–100 nm) [31] and are modified by being dispersed in various stabilizers such as citrate buffer, phosphate-buffered saline (PBS), H_2_O, and D_2_O [86]. This is undertaken to produce AuNPs with different shapes, sizes, and stabilization media [87]. However, some of these modifications result in more unstable nanoparticles over time, and some have a significant effect on the size distribution and potentially on the shape of the particles [86]. Suitably modified AuNPs provide excellent stability and biocompatibility, which makes them suitable for the detection of analytes in heavy metals or complex biological systems [88,89]. For the detection of analytes, AuNPs can be produced by three synthesis methods: physical, chemical, and biological. The characteristics of the AuNPs produced by each synthesis method are described in Table 2.

Based on the research in Table 2, the AuNPs that are commonly produced using these synthesis methods are around 1–40 nm. There are several shapes produced from the synthesis of AuNPs, but the spherical shape is the most common form of AuNP produced. In the sonochemical method, the size of the AuNP decreases with an increasing sonication output power, which means that the surface plasmon resonance, particle size, stability, and monodispersity of AuNPs depend on the ultrasonic output power and reaction time [74]. In the photochemical method, a longer incubation time can increase the size of AuNPs [93]. In the seeded growth method, the concentration of the reducing agent plays a role in the particle size of AuNPs [77]. In the Turkevich method, the influence of molar ratios, reagent concentrations, and temperature in the synthesis process plays a role in the characteristics of AuNPs [78]. In the microorganism synthesis method, different microorganisms produce nanoparticles with different sizes and shapes [97].

The different synthesis methods do not show significant differences in the size of AuNPs. However, certain critical conditions in the synthesis process such as the incubation time, reagent concentration, temperature, and different microorganisms can affect the different characteristics of AuNPs.

### 3.3. Conjugation of AuNPs

AuNPs can be conjugated with various molecules to provide specific functions in certain applications.

#### 3.3.1. DNA-Conjugated AuNPs

DNA molecules can be attached to AuNPs. These conjugates are used in DNA-based sensing applications such as DNA hybridization assays or gene detection. DNA–AuNPs can enable the colorimetric, fluorescent, or electrochemical detection of specific DNA sequences [100].

#### 3.3.2. Protein-Conjugated AuNPs

Proteins can be conjugated to AuNPs for various applications including biosensing, drug delivery, or catalysis. The protein can provide specific binding capabilities or catalytic activity, while the AuNPs can enhance the stability [101].

#### 3.3.3. Aptamer-Conjugated AuNPs

Aptamers are synthetic oligonucleotides or peptides with a high affinity and specificity for target molecules. Aptamer–AuNP conjugates are used for biosensing, drug delivery, or therapeutics as they can selectively bind to their target molecules [24].

## 4. Application of Detection Strategies Based on AuNPs

Noble metal nanoparticles, especially AuNPs, are at the forefront of nanomaterials research because of their excellent size dependence and optical properties. Controlling the shape and size of nanoparticles during synthesis is critical to obtain the optimal nanosystem for any given application [102]. AuNPs show excellent selectivity and sensitivity as colorimetric sensors when these are mostly of spherical morphology [103].

### 4.1. Application of AuNPs for the Detection of Heavy Metals

Metal nanoparticles, especially AuNPs, have been developed for the colorimetric detection of many heavy metal ions. Successful integration of these nanomaterials for the selective detection of heavy metals first requires functionalizing the surface of AuNPs with receptors without altering the stability of the functional groups in solution. The sensitivity and selectivity of the system are determined by precise ligand selection and design [20]. The application of AuNPs in heavy metal detection is summarized in Table 3.

Based on research by Megarajan et al. (2020), rapid colorimetric sensing of Pb^2+^ ions in water samples induces the aggregation of N-decanoyltromethamine (NDTM)-capped AuNPs. The AuNPs formed with NDTM is highly stable; the color change from pink to violet in the presence of Pb^2+^ is caused by the aggregation state of NDTM-AuNPs. The morphology of the NDTM-AuNPs can be measured using a high-resolution transmission electron microscope (HR-TEM). AuNPs bound to NDTM produce an average size of 29 ± 7 and have a spherical shape. The color change of NDTM-AuNPs can be attributed to destabilization by Pb^2+^ ions. The moment Pb^2+^ was added to NDTM-AuNPs, it was shown that the average size of the NDTM-AuNPs increased to 57.5 nm in the presence of Pb^2+^ ions.

Nguyen et al. (2022) discussed AuNPs stabilized on poly(styrene-co-maleic anhydride) (PSMA) as an effective colorimetric detection of Pb(II). PSMA with reactive anhydride groups that are easily modified can be used as a supporting polymer for various catalysts. AuNPs stabilized on PSMA (Au@PSMA) can be used as a colorimetric probe to detect heavy metal ions. The average size of Au@PSMA was ±50 nm and was spherical in shape. In the absence of PSMA, Au^3+^ ions were evenly dispersed in an aqueous solution. In the presence of PSMA, Au^3+^ ions gathered around the COO- groups of PSMA and formed larger particles. The detection limit of analysis was 30 nM.

Azzam et al. (2014) enhanced the detection of Ni^2+^ and Zn^2+^ ions using a nanostructure of synthesized dithiol surfactants with AuNPs. The TEM technique was used to investigate the stabilization of AuNPs and the self-assembling of these surfactants on AuNPs with the synthesized dithiol surfactants and showed that the average particle size of the AuNP-dithiol surfactants was 20 nm and had a spherical shape. After the addition of the dithiol surfactant, the AuNPs also showed similar particle diameters but were more dispersed than a single AuNP due to the presence of alkyl chains and the formation of nanoshells, which reduced the aggregation of AuNPs.

Li et al. (2020) discussed a sensitive and selective colorimetric detection of arsenic(III) based on glutathione (GSH)-functionalized AuNPs. In the GSH-AuNP solution, a bond will occur between As^3+^ and GSH if As^3+^ ions are added to the solution; the bond will induce the aggregation of AuNPs. The aggregation of GSH/AuNPs serves as indicator of As^3+^ ions, with a perceptible color change from wine red to blue. Then, the GSH solution is added to the AuNP solution, thus obtaining GSH ligand-functionalized AuNPs. The size and morphology of AuNPs were characterized by TEM and the diameter of the AuNPs was 40 nm.

Zhang et al. (2022) discussed the determination of As(III) based on negatively-charged aptamer-mediated aggregation of positively-charged AuNPs. The advantage of colorimetric methods based on positively-charged AuNPs is that aggregation can be regulated by a negatively-charged aptamer without the addition of salts. TEM was used to observe the degree of aggregation of (+)AuNPs, where the average diameter of (+)AuNPs was about 43 nm.

Amanulla et al. (2018) assembled sulfur-doped graphitic carbon nitride (S-g-C_3_N_4_) on chitosan-functionalized AuNPs for the colorimetric detection of trace Hg^2+^. Graphitic carbon nitride was used to reduce the limitations of AuNPs. The currently reported carbon-based nanoparticles have the advantageous properties of sensor systems with high sensitivity and selectivity. After the addition of mercury, the LSPR of the AuNPs/S-g-C_3_N_4_ showed a significant blue shift, causing the solution to change color from wine red to colorless due to a change in the morphology of AuNPs/S-g-C_3_N_4_ through the formation of an Au–Hg amalgam. The HR-TEM image showed that the size of the AuNP-S-g-C_3_N_4_ was around 30 nm with a spherical shape. Several small black spots indicated the presence of AuNPs decorating the surface of S-g-C_3_N_4_.

Liu et al. (2023) enhanced the simple visual and rapid colorimetric detection of Hg^2+^ in cosmetics based on gold nanoparticles modified by sulfadiazine. The addition of sulfadiazine did not change the state of the AuNP dispersion and the color change of the AuNP solution. When Hg^2+^ was added to the solution, the pyridyl and –SO_2_ groups of the sulfadiazine-AuNP system were chelated with mercury ions and induced the AuNP aggregation. The size of the sulfadiazine-AuNPs was about 15 nm with a spherical shape. The average size of the AuNPs increased significantly in the presence of Hg^2+^.

Bhamore et al. (2021) discussed the functionalization of AuNPs using guanidine thiocyanate (GT) for the detection of Cd^2+^. GT can form a self-assembled monolayer on the surface of AuNPs. In addition, the negative charge on the GT surface provides a repulsive force that prevents the self-aggregation of AuNPs. The addition of Cd^2+^ stimulates the chelation of GT-AuNPs, through which the destabilization of AuNPs occurs and hence agglomeration. The TEM images showed that the AuNPs were spherical, well-dispersed, and uniform with an average size of 17.5 ± 3.5 nm. The GT-modified AuNPs were highly stable at 4 °C for up to 3 months.

Shellaiah and Sun (2022) performed research on cysteamine-functionalized nanodiamond (NDC) conjugated to AuNPs to deliver NDC-AuNPs that were utilized in the enhanced colorimetric detection of Cr^3+^. The NDCs were conjugated over the bare AuNP surface to form NDC-AuNPs. The initial and mild NDC-AuNP aggregation took place at pH 6 and showed rapid aggregation in the presence of Cr^3+^ ions. The formation of NDC-AuNPs is well-recognized, with an average particle size of 18.3 ± 4.8 nm and a spherical shape.

Ejeta and Imae (2021) enhanced the selective colorimetric detection of the Cr(III) pollutant in water on 3-mercaptopropionic acid (3-MPA) functionalized gold plasmon nanoparticles. The average size of the AuNPs was 16.6 ± 2.3 nm. When the ligand covered the AuNPs, the average size of AuNPs-3-mpa became 17.1 ± 2.5 nm with a spherical shape. After the addition of Cr(III), the average size increased to 24.2 ± 4.4 nm. This shows that the size of AuNPs will increase in the presence of Cr(III), and aggregation will occur.

Borah et al. (2022) enhanced the highly selective, rapid, and simple colorimetric detection of Fe^3+^ in fortified foods by L-cysteine modified AuNPs (Cys-Caf-AuNPs). Regarding the addition of Fe^3+^ to Cys-Caf-AuNPs, the colloidal solution changed from dark ruby red to colorless upon the addition of the analyte. This was due to the effective binding interaction of the Cys-Caf-AuNP to Fe^3+^. The TEM image showed that the size of the Cys-Caf-AuNPs was about 22.02 ± 1.5 nm with a spherical shape.

Andreani et al. (2021) discussed a fast and selective colorimetric detection of Fe^3+^ based on gold nanoparticles capped with ortho-hydroxybenzoic acid (o-HBA). o-HBA contains carboxylate and hydroxyl phenolic functional groups that would be suitable to act as capping and reducing agents. The presence of Fe^3+^ caused the size of the o-HBA-AuNPs to grow larger, changing the SPR spectra, and inducing a color change from red to dark purple. The size of the o-HBA-AuNPs after the addition of Fe^3+^ was analyzed by DLS, which showed an increase from 25.04 nm to 651.50 nm and induced aggregation.

As shown in Table 3, AuNPs have been widely applied in heavy metal detection. The conjugation used in each study improved the stability of AuNPs in the detection process. The AuNP particles obtained after conjugation had a variety of sizes. The particle size observation was carried out using TEM/HR-TEM. Based on the research shown in Table 3, the particles used for Hg^2+^ detection were the smallest, with a value of ±15 nm; the largest were those used for Pb^2+^ detection, with a size of 50 nm. In general, AuNPs used in detecting heavy metals can range in particle size, around 15–50 nm. The shape that is often found is a spherical shape. Based on the research, a spherical shape is a common shape in AuNPs, and the particle size depends on the conjugate used in the detection method. The LOD obtained also has various values, and the resulting value depends on the conjugation that occurs between the AuNPs and the conjugate.

### 4.2. Application of AuNPs for the Detection of Biological Molecules

In the fields of biosensor technology and diagnostics, nanoparticles are usually used as probes, functionalized and conjugated with biological molecules. Bioconjugation involves an interaction of the chemical/functional groups from the nanoparticle and the biomolecule. The interaction depends on the type of molecules used, which can be physical or chemical. Interactions between nanoparticles and biomolecules can be enhanced by incorporating various ligands on the interacting surfaces, which improves the stability of the interactions [41]. The application of AuNPs in biological molecule detection is summarized in Table 4.

Rawat et al. (2017) discussed the detection of spermine and spermidine in biological samples by tyrosine-protected AuNPs for colorimetric sensing. Tyrosine was chosen as a multifunctional ligand to fabricate AuNPs and their specific interactions with spermine and spermidine, allowing for the sensitive detection of target analytes with minimal sample volume. The Tyr-AuNPs showed a strong surface affinity toward spermine and spermidine via electrostatic interactions and hydrogen bonding. The absorbance changes were measured upon the addition of spermine and spermidine. The TEM images of Tyr-AuNPs revealed that they were roughly spherical and well-dispersed with an average size of 10.2 ± 3.3 nm.

Loganathan et al. (2021) used 2-amino-4-thiazoleacetic acid (ATA)-conjugated AuNPs for the detection of quinalphos (QP). The detection mechanism followed the aggregation of ATA-AuNPs after interacting with QP. TEM images showed that the average particle size of the ATA-AuNPs was 6.4 nm with a chain-like morphology. The interaction of QP with ATA-AuNPs caused aggregation, which in turn changed the color of the colloidal solution to purple from wine red. The colloidal ATA-AuNP solution was highly stable for more than 6 months.

Tu et al. (2023) discussed a colorimetric aptasensor based on aptamer–polythymine (polyT)–polyadenine (polyA)–AuNPs (pA–pT–apt@AuNPs) for the detection of amyloid-β oligomers. The polyA block can be preferentially attached to the surface of AuNPs and the aptamer sequence was used for specific target recognition. PolyT makes the aptamer block upright and facilitates the recognition of protein targets. pA–pT–apt@AuNPs are colloidally stable and the solution remains red in color at a salt concentration of 150 mM NaCl. TEM confirmed the formation of AuNPs that were 13 nm in diameter. In quantitative detection, as the concentration of amyloid-β oligomers increases, the aggregation degree of AuNPs decreases.

Khan and Park (2021) enhanced a new sensitive colorimetric method for the detection of Aβ_1–40_. Sequential binding of Aβ_1–40_ to AuNPs and metal ions was designed and tested to determine Aβ-specific AuNP aggregation and generate quantitative colorimetric signals. The average diameter of the synthesized AuNPs observed by TEM was 24 ± 1 nm. This indicates that all AuNP binding sites were occupied by Aβ_1–40_. Similarly, the average size of the AuNPs was increased to 28 ± 1.2 nm. The increase in size indicates the conjugation of Aβ_1–40_ on the AuNPs and Ni-HRP on AuNP–Aβ_1–40_.

Chen et al. (2020) carried out colorimetric sensing of histidine by N-acetyl-cysteine (NAC)-functionalized AuNPs in biological samples. NAC can be modified on the AuNP surface via the Au–S covalent bond. The carboxyl group and amino group in the structure can dissociate in solution. The NAC-AuNP sensor was synthesized to realize the sensitive detection of histidine under the optimal external conditions (pH value of 6, temperature of 20 °C); the particles remained stable in the solution. In the presence of histidine, the color of the NAC-AuNP solution changed from yellowish pink to grey while the characteristic absorption peak red-shifted from 515 nm to 550 nm. The TEM image showed that the NAC-AuNPs were uniformly dispersed in the aqueous solution with an average diameter of about 6 nm and a spherical shape.

Zhuang et al. (2021) enhanced a colorimetric sensor for the sensitive detection of tyrosinase activity based on 4-mercaptophenyl boronic acid (4-MPBA) modified AuNPs. Tyramine (Tyr) was used to modify the MBs surface using a covalent bond between the amino groups of tyramine and the carboxyl group of the magnetic beads. 4-MPBA-AuNPs adsorbed on the surface of tyramine MBs pretreated with Tyr and ascorbic acid, leading to an obvious color change in the supernatant. TEM image results showed that the diameter of the 4-MPBA-AuNPs was about 30 nm.

Mirsalari and Elhami (2020) enhanced the colorimetric detection of insulin in human serum using a GO/AuNPs/TX-100 nanocomposite. In the presence of sodium citrate, AuNPs were formed from chloroauric acid by growing on GO nanosheets. This occurred due to the electrostatic interaction between AuNPs and the oxygen groups of the graphene material. The increase in the size of AuNPs through conjugation to the GO confirmed that AuNPs were assembled on the GO. This result is consistent with the TEM images, which showed the dominant average particle size of 15–20 nm. However, there would be a change in size to become larger when insulin is added to the solution.

Dadmehr et al. (2022) discussed a colorimetric detection of matrix metalloproteinase MMP-9 as a cancer biomarker based on a AuNPs@gelatin/AuNC nanocomposite. In order to use the spherical AuNPs as the detecting platform, the produced gelatin/AuNCs were coated and deposited around them. The TEM image revealed the presence of the gelatin/AuNC layer surrounding the spherical AuNPs, which had an average size of 18 nm. The aggregation condition of the AuNPs was produced by the gelatinase activity of the MMP-9 enzyme. Upon the addition of various concentrations of the MMP-9 enzyme, the visual detection of the samples’ color ranged from red to purple.

Cheng et al. (2022) discussed whether temperature modulating the peroxidase-mimic activity of poly(N-isopropyl acrylamide) (PNIPAM) protected the gold nanoparticles for the colorimetric detection of glutathione. The particle size distribution of PNIPAM@AuNPs was an average of 6.4 ± 0.6 nm. Particularly, the addition of GSH significantly reduced the reaction solution of the blue color.

As shown in Table 4, she shape of the resulting AuNPs also generally remained spherical, either homogeneously or well-dispersed. The smallest particle size shown was 6 nm in the detection of histidine. The largest particle size was 28–35 nm in the detection of amyloid-β oligomers. Based on research, the average particle size commonly used to detect biological molecules is in the range of 6–35 nm. The size range of AuNPs produced in biological molecules is not larger than the AuNPs used in heavy metal detection applications. The LOD results also vary depending on the conjugate used.

### 4.3. Application of AuNPs for the Detection of Pharmaceutical Compounds

The application of AuNPs in the detection of pharmaceutical compounds is summarized in Table 5.

Khurana et al. (2020) designed a colorimetric sensing strategy for pharmaceutical compounds based on citrate-reduced AuNPs for the detection of sanguinarine (an anticancer drug). The AuNPs were prepared by the Turkevich method using trisodium citrate and are also known as unmodified AuNPs because they were not functionalized with another conjugate. The electrostatic interaction between sanguinarine and AuNPs induced the aggregation of the AuNPs, which was accompanied by a visible color change in the colloidal solution from red to blue. The LOD was calculated to be 46 nM. The AuNPs were found to be spherical in shape, having an average size of 25–30 nm.

Zou et al. (2020) discussed a colorimetric aptasensor for the detection of kanamycin based on catalytic hairpin assembly amplification and DNA-AuNP probes. For the detection of antibiotics, aptasensors, or aptamer-based biosensors, have emerged as a possible substitute for current techniques. Up to now, numerous aptasensors have been created for the detection of antibiotics by utilizing a variety of methods such as colorimetric. In the presence of kanamycin, the hairpin probes (H1, H2, and H3) can form branched Y-shaped DNA nanostructures that possess three single-strand DNA (ssDNA) tails, which induce the assembly of DNA-AuNPs, resulting in a concomitant color change from red to blue-purple. The proposed aptasensor showed high sensitivity and selectivity for kanamycin detection with the LOD calculated to be 0.01 nM. TEM measurements oof the morphology of the particles showed a well-dispersed and almost spherical shape with an average diameter of about 13 nm.

Qi et al. (2021) enhanced a label-free colorimetric aptasensor based on split aptamers-chitosan oligosaccharide-AuNP nanocomposites for the sensitive and selective detection of kanamycin. Under acidic conditions, the split aptamers containing polyA sequences were absorbed onto the surface of AuNPs via the ployA–Au interaction to create the split aptamer–AuNP complex. In the presence of kanamycin, the split aptamers preferentially bonded to kanamycin, leading to apparent aggregation. TEM imaging showed that the AuNPs were spherical, with an average size of about 25.3 nm.

Xu et al. (2022) discussed target-induced gold nanoparticle colorimetric sensing coupled with an aptamer for the rapid and high-sensitivity detection of kanamycin. Kanamycin is positively charged, and AuNPs are negatively charged, so when AuNPs are coated with the aptamer, AuNPs with the addition of kanamycin could exhibit a color change. This indicates that kanamycin could also aggregate aptamer-coated AuNPs, even in the absence of any other inducing reagent. TEM characterization of the aptamer-coated AuNPs showed that they were uniformly dispersed in the natural state, and were approximately spherical in shape with an average size of 13 nm.

Nguyen and Jang (2022) developed a colorimetric aptasensor for the detection of amoxicillin based on the Tris-HCl buffer-induced aggregation of AuNPs. Because aptamers can bind target molecules with high affinity and specificity, aptamers are frequently utilized as recognition components to increase the specificity of biosensors. By increasing the electrostatic repulsion between the nanoparticles, the unfolded aptamers can easily adsorb onto the negatively charged surfaces of AuNPs. This prevents the AuNPs from salt-induced aggregation. Tris-HCl buffer showed some of benefits including the ability to regulate the pH of the solutions as well as the aggregation of AuNPs. This resulted in the aggregation of AuNPs by Tris-HCl buffer, and the color of the solution containing AuNPs changed from red to blue with a LOD of 0.067 nM. The results were confirmed by TEM and the AuNPs had a spherical morphology, a mean diameter of 13 nm, and were stable in aqueous solution.

Zhang et al. (2020) researched a simple and sensitive colorimetric sensor for the determination of gentamicin in milk based on lysine-functionalized AuNPs. The average particles of the lysine-AuNPs were 13 nm and were in a stable solution. The functionalization of the AuNPs was completed by the addition of lysine to the AuNPs solution. Gentamicin caused the AuNPs to polymerize after it was introduced, changing the hue of the solution as a result of the strong hydrogen connection between gentamicin and lysine. Utilizing the difference in color before and after AuNP aggregation, gentamicin could be quantitatively detected. The interaction between lysine and AuNPs caused lysine to be adsorbed on the surface of the AuNPs. The addition of gentamicin promoted the aggregation of AuNPs due to strong hydrogen bonding between gentamicin and lysine. The AuNP color changed from wine red to blue. The results showed that the LOD was 1.22 nM.

Wu et al. (2019) discussed a label-free colorimetric aptasensor based on the controllable aggregation of AuNPs for the detection of multiplex antibiotics (chlorampenicol and tetracycline). Preventing surface modification and mediating regulated AuNP aggregation in high ionic systems, the aptamer acted as a molecular switch. The AuNP solution simultaneously displayed color changes with the signal readout. In the presence of chlorampenicol and tetracycline, the aptamer recognition fragment detached from the surface of the AuNP and bonded strongly to the target to form a folded rigid structure. At the same time, the non-recognition part of aptamer could not maintain the stability of the AuNPs, leading to aggregation. The TEM image showed that the prepared AuNPs were spherical with a diameter of about 12 nm.

Xiao et al. (2022) enhanced a simple and sensitive AuNP-based colorimetric aptasensor for the specific detection of azlocillin. The azlocilin detection method was developed using the DNA aptamer as a recognition element and unmodified AuNPs as the colorimetric indicator. When azlocillin is present, aptamers specifically bind to azlocillin and lose their ability to shield AuNPs from aggregation caused by NaCl. This weakens the absorption peak of the AuNPs and causes the solution’s color to change, allowing for the quantitative analysis of azlocillin. The size of the AuNPs determined by TEM was approximately 13 nm.

In the detection of pharmaceutical compounds, AuNP conjugation also provides good stability with the conjugate. As shown in Table 5, the AuNPs formed also commonly produce spherical shapes. Based on the research, the AuNPs had the same size as that shown in several studies that have been carried out, of around 13 nm. The smallest particles size shown was 12 nm in the detection of chloramphenicol and tetracycline. The largest particle size was 25–30 nm in the detection of sanguinarine. The LOD also showed a sensitive value and was analyzed with the naked eye. The conjugation of AuNPs with conjugates decreased the LOD value for pharmaceuticals, making the AuNP sensor more sensitive.

## 5. Conclusions and Future Perspective

AuNPs have been developed and used in various fields. AuNPs have been modified with various conjugates to increase the sensitivity, selectivity, and stability for each desired application. The synthesis and conjugation methods are important factors in determining the particle size, one of the factors that needs to be considered in the application method. AuNPs used in heavy metal detection generally have a particle size of around 15–50 nm. In the detection of biological molecules, the particle size of AuNPs commonly used is 6–35 nm, while in the detection of pharmaceutical compounds for cancer treatment or other drugs, the particle size used is generally 12–30 nm. Each of these particle sizes is often and widely used in every detection method and also provides the best results in the detection method. The particle sizes did not correlate with the type of molecules regardless of whether they were heavy metals, biological molecules, or pharmaceutical compounds, but depended on the properties of the molecule itself. In addition to choosing the size of the AuNP, the shape of the AuNP is also an important factor. In general, the most common morphology found in both the synthesis and conjugation with other compounds was a spherical shape. Furthermore, to obtain a form of AuNP that is stable for use, functionalization can be carried out with a conjugate/receptor to increase the stability of both the AuNP itself and increase the solubility to achieve the maximum detection results.

In future research, more modifications and the conjugation of AuNPs with other conjugates still need to be explored in order to provide detection methods that are more sensitive, selective, and stable. Adjusting or incorporating a smartphone application with the sensor being made could be explored to enhance the sensitivity of the sensor.

## Figures and Tables

**Table 1:** Comparison of the physical, chemical, and biological methods in the synthesis of AuNPs [62,69,70,71].

Physical Methods	Chemical Methods	Biological Methods
No use of toxic chemicals	Use of toxic chemicals	No use of toxic chemicals
Difficult to control size and shape	Easy to control size and shape	Easy to control size and shape
Low stability	High stability	Low stability

**Table 2:** Characteristics of the AuNPs in each synthesis method.

Synthesis	Method	Characteristics of AuNPs	Reference
Physical	Laser Ablation	Average size of 8 nm with spherical shape	[64]
		Size ranging between 10 and 25 nm with spherical shape	[73]
		Average size of 10 ± 2 with a predominantly spherical shape (some irregular shapes also observed)	[90]
	Sonochemical	Size ranging between 13.6 and 22.3 nm with a spherical shape	[74]
		Average size of 8.7 nm with a spherical shape	[91]
		Average size of 21 nm with a spherical shape	[92]
	Photochemical	Average size of 10 nm with a spherical shape	[93]
		Average size of 11 nm with a dominantly spherical shape	[94]
Chemical	Seeded Growth	Average size of 27 nm with a spherical shape	[78]
		Size ranging between 5.09 ± 17.22 nm with a spherical shape	[95]
	Turkevich	Average size of 15 nm with a uniform spherical shape	[62]
		Size ranging between 15 and 30 nm with a spherical shape	[78]
		Average size of 17.9 ± 3.6 nm with a similar quasi-spherical morphology	[96]
Biological	Microorganism	Size ranging between 20 and 40 nm with a spherical shape	[97]
		Size ranging between 7 and 21 nm with a spherical shape	[66]
		Average size of 15 nm with a spherical shape	[98]
	Plant Extract	Size ranging between 1.3 and 15.6 nm with a spherical and triangle shape	[61]
		Size ranging between 2.65 and 16.25 nm with a circular shape	[67]
		Average size of 32.96 ± 5.25 nm with a spherical shape	[99]

**Table 3:** Application of AuNPs in the detection of heavy metals.

Analytes	Conjugated AuNPs	Size and Shape of AuNPs	LOD	Stability	Reference
Pb^2+^	N-decanoyl-tromethamine	29 ± 7 nm with a spherical shape	350 nM	Highly stable	[17]
	Poly(styrene-co-maleic anhydride)	±50 nm with a spherical shape	30 nM	Well stable	[104]
Ni^2+^, Zn^2+^	Dithiol surfactants	±20 nm with a spherical shape	-	Well stable	[18]
As^3+^	Glutathione	±40 nm with a spherical shape	0.11 ppb	Well stable	[19]
	Cysteamine	±43 nm with a spherical shape	7.41 nM	Stable (6 days)	[105]
Hg^2+^	S-g-C_3_N_4_	±30 nm with a spherical shape	0.275 nM	Highly stable	[16]
	Sulfadiazine	±15 nm with a spherical shape	71 ppb	Stable	[106]
Cd^2+^	Guanidine thiocyanate	17.5 ± 3.5 nm with a spherical shape	10 nM	Highly stable	[20]
Cr^3+^	Cysteamine	18.3 ± 4.8 nm with a spherical shape	0.236 nM	Highly stable	[21]
	3-merca-ptopropionic acid	17.1 ± 2.5 nm with a spherical shape	0.34 ppb	Stable	[107]
Fe^3+^	L-Cysteine	22.02 ± 1.5 nm with a spherical shape	220 nM	Highly stable	[108]
	ortho-hydroxybenzoic acid	±25.04 nm with a spherical, triangular, and hexagonal shape	9190 nM	Highly stable	[109]

**Table 4:** Application of AuNPs in the detection of biological molecules.

Analytes	AuNP Conjugates	Size and Shape of AuNPs	LOD	Stability	Reference
Spermine and spermidine	Tyrosine	10.2 ± 3.3 nm with a roughly spherical shape and was well-dispersed	0.136 and 0.636 nM	-	[22]
Quinalphos	2-Amino-4-thiazoleacetic acid (ATA)	±6.4 nm with a spherical shape	48.2 nM	Highly stable	[23]
Amyloid-β (Aβ) oligomers	Aptamer–polythymine–poly-adenine	±13 nm and a spherical shape	3.03 nM	Well stable	[24]
	Aβ-Nickel-horseradish peroxidase (Aβ-Ni-HRP)	28–35 nm with a spherical shape	0.22 nM	-	[110]
Histidine	N-Acetyl-cysteine	±6 nm with a spherical shape and was well-dispersed	0.167 nM	Stable	[25]
Tyrosinase	4-mercaptophenyl boronic acid (4-MPBA)	±30 nm with spherical shape	0.001 U/mL	-	[111]
Insulin	Graphene oxide and triton X-100	±15–20 nm with a dominant spherical shape	0.1 ppb	Stable	[112]
Matrix metalloproteinase (MMPs)	Gelatin/AuNCs	±18 nm with a spherical shape	0.25 ppb	Well stable	[113]
Glutathione (GSH)	Poly(N-isopropyl acrylamide) (PNIPAM)	6.4 ± 0.6 nm with a spherical shape	800 nM	High stable	[114]

**Table 5:** Application of AuNPs in the detection of pharmaceutical compounds.

Analytes	AuNP Conjugates	Size and Shape of AuNPs	LOD	Stability	Reference
Sanguinarine	-	±25–30 nm with spherical shape	46 nM	Stable	[115]
Kanamycin	DNA (hairpin probes H1, H2, H3)	±13 nm with almost spherical shape	0.01 nM	-	[116]
	Poly A-split aptamer kanamycin	±25.3 nm	20.58 nM	High stable	[117]
	-	±13 nm with spherical shape	4 nM	Stable	[118]
Amoxicillin	Aptamer and Tris-HCl buffer	±13 nm with spherical shape	0.067 nM	Well stable	[119]
Gentamicin	Lysine	±13 nm	1.22 nM	Stable	[120]
Chloram-phenicol and tetracycline	-	±12 nm with spherical shape	7 nM and 32.9 nM	Stable	[121]
Azlocillin	-	±13 nm	11.6 nM	Stable	[122]

## Data Availability

Data sharing is not applicable.
